# Reconsidering dopaminergic modulation in Alzheimer's disease: A case for levodopa/carbidopa as a disease‐modifying agent

**DOI:** 10.1002/alz.70532

**Published:** 2025-07-22

**Authors:** Annalisa Nobili, Marcello D'Amelio

**Affiliations:** ^1^ Department of Medicine and Surgery Università Campus Bio‐Medico di Roma Rome Italy; ^2^ Department of Experimental Neurosciences IRCCS Santa Lucia Foundation Rome Italy

**Keywords:** biomarkers, cognitive decline, dopamine, levodopa/carbidopa, ventral tegmental area

1

Dear Editor,

We are writing this letter in response to the article by Sárkány et al. recently published in *Alzheimer's & Dementia*.^1^

The study titled “Association between the use of levodopa/carbidopa, Alzheimer's disease biomarkers, and cognitive decline among participants in the National Alzheimer's Coordinating Center Uniform Data Set” reignites a long‐standing yet controversial question: Can restoring dopaminergic tone modify Alzheimer's disease (AD) progression?

Leveraging a large cohort of 1942 subjects with available cerebrospinal fluid (CSF) biomarker data and 20,348 person‐years of longitudinal follow‐up, the authors applied robust statistical methodology, including propensity score matching for age, sex, and apolipoprotein E ε4 status. Their methodological strength is further enhanced by the use of mixed‐effects models and negative control comparators—namely, the users of non‐levodopa antiparkinsonian agents.

The analysis reveals that levodopa/carbidopa (LD/CD) use is associated with reduced CSF concentrations of amyloid beta (Aβ)₄₂, total tau (t‐tau), and phosphorylated tau (p‐tau), across diagnostic categories, including cognitively normal (NC), mild cognitive impairment (MCI), and dementia (DE) groups. Notably, the LD/CD use does not influence biomarker levels in subjects treated with dopamine agonists, suggesting a mechanism dependent on presynaptic dopamine replenishment rather than postsynaptic receptor activation.

Clinically, LD/CD use was associated with a median delay of 2.1 years in the progression from MCI to DE. However, no significant impact was observed on the transition from NC to MCI. Importantly, a safety signal emerged: increased all‐cause mortality among cognitively normal LD/CD users. This finding raises critical concerns regarding off‐label or prophylactic use and warrants further investigation into possible autonomic or synucleinopathy‐related mechanisms.

Mechanistically, these findings challenge current interpretations of biomarker dynamics. While decreased CSF Aβ₄₂ is typically considered a surrogate for increased cortical amyloid deposition,^2^ the concurrent attenuation of tau pathology and slower clinical progression suggest alternative mechanisms. Preclinical evidence proves that long‐term LD treatment improves Aβ pathology and memory function in AppNL‐F—a validated mouse model of AD—through increased neprilysin expression.^3^ Moreover, dopamine has been shown to inhibit glycogen synthase kinase‐3β (GSK‐3β), thereby attenuating tau hyperphosphorylation.^4^ These two complementary mechanisms—Aβ clearance and tau modulation—may explain the observed biomarker profile. The absence of similar effects with dopamine agonists, which do not replenish intracellular dopamine stores, underscores the specificity of LD/CD's mode of action.

This study also aligns with growing evidence that early degeneration of dopaminergic circuits—particularly the ventral tegmental area (VTA)^5^, ^6^ and mesocorticolimbic pathways—precedes cortical amyloidosis^5^ and may contribute to both the neuropsychiatric and cognitive symptoms observed in prodromal AD.^7^, ^8^ Supporting this, recent preclinical data^9^ demonstrate that repetitive prefrontal transcranial direct current stimulation re‐engages residual VTA dopaminergic neurons in an AD mouse model, thereby mitigating synaptic, cognitive, and neuroinflammatory AD‐like deficits and further substantiating the therapeutic relevance of this circuit. Given its ability to cross the blood–brain barrier and restore central dopaminergic tone, LD/CD may uniquely engage this underappreciated axis of AD pathophysiology.

Nonetheless, important limitations remain. Treatment heterogeneity across study centers—including variation in LD/CD dosage, duration, and Parkinsonian symptomatology—complicates the interpretation of both biomarker and clinical outcomes. Furthermore, only a subset of the cohort fulfilled contemporary amyloid/tau/neurodegeneration biomarker criteria, limiting generalizability to amyloid‐confirmed populations. The increased mortality observed in the NC subgroup is particularly concerning and may reflect either prodromal Lewy body pathology or LD/CD‐induced autonomic dysregulation.

In light of these findings, a biomarker‐enriched, phase II randomized controlled trial of low‐dose LD/CD in prodromal AD is both warranted and timely. Such a trial should incorporate longitudinal assessments of CSF and plasma proteomics, amyloid‐ and tau‐positron emission tomography (PET) imaging, and dopaminergic PET ligands to confirm central target engagement. Parallel in vitro investigations should explore LD/CD's effects on neprilysin activity, lysosomal–autophagic flux, and microglial polarization. Stratification by dosage, treatment duration, baseline biomarker status, and motor symptom burden—alongside vigilant safety monitoring—will be essential to assess both efficacy and tolerability.

In an era increasingly dominated by costly biologics that target surrogate endpoints of uncertain clinical validity, the work by Sárkány et al. offers a disruptive and compelling alternative: that a long‐approved, orally administered dopaminergic agent may exert mechanistically grounded, disease‐modifying effects in AD. Unlike monoclonal antibodies, LD/CD's therapeutic effects are not solely predicated on Aβ clearance, thus sidestepping the scientific and regulatory concerns recently highlighted^10^ regarding the premature adoption of unvalidated biomarker endpoints. If prospectively validated, these findings may mark a paradigm shift—from targeting pathology downstream to reinforcing the resilience of vulnerable neural circuits.

## AUTHOR CONTRIBUTIONS

Conceptualization and writing: Annalisa Nobili and Marcello D'Amelio.

## CONFLICT OF INTEREST STATEMENT

The authors declare no conflicts of interest. Author disclosures are available in the supporting information.

## Supporting information



Supporting Information
